# Does ENaC Work as Sodium Taste Receptor in Humans?

**DOI:** 10.3390/nu12041195

**Published:** 2020-04-24

**Authors:** Albertino Bigiani

**Affiliations:** Dipartimento di Scienze Biomediche, Metaboliche e Neuroscienze, Università di Modena e Reggio Emilia, 41125 Modena, Italy; albertino.bigiani@unimore.it; Tel.: +39-059-205-5349

**Keywords:** sodium taste, sodium receptor, salt taste, amiloride, taste transduction

## Abstract

Taste reception is fundamental for the proper selection of food and beverages. Among the several chemicals recognized by the human taste system, sodium ions (Na^+^) are of particular relevance. Na^+^ represents the main extracellular cation and is a key factor in many physiological processes. Na^+^ elicits a specific sensation, called salty taste, and low-medium concentrations of table salt (NaCl, the common sodium-containing chemical we use to season foods) are perceived as pleasant and appetitive. How we detect this cation in foodstuffs is scarcely understood. In animal models, such as the mouse and the rat, the epithelial sodium channel (ENaC) has been proposed as a key protein for recognizing Na^+^ and for mediating preference responses to low-medium salt concentrations. Here, I will review our current understanding regarding the possible involvement of ENaC in the detection of food Na^+^ by the human taste system.

## 1. Introduction

The sodium ion (*Na*^+^) is an essential mineral for our body because it regulates the osmolality of the extracellular fluid and plays a key role in many physiological processes, from the generation of nerve impulses to renal function. Na^+^ is lost continuously through urine, feces, and sweat. Thus, to maintain proper bodily balance, we need to replace losses by the ingestion of food containing this cation. Our ability to detect Na^+^ in foodstuffs relies on the taste system: Na^+^ elicits a specific sensation called salty taste [1,2] that guides the intake of this important mineral [3,4]. Table salt (NaCl) represents a prototypic chemical substance evoking salty taste. It is well established that Na^+^ is responsible for the perceived saltiness and for the pleasantness of low to medium concentrations of table salt [4,5].

The early events in taste reception typically include the interaction of the chemical stimulus with a membrane receptor in taste cells, specialized epithelial cells clustered in sensory end-organs called taste buds [6]. Most of studies on sodium taste reception have been performed on laboratory rodents (mouse and rat). In these mammals, the epithelial sodium channel (ENaC) works as low-salt receptor, mediating acceptance responses to low-medium salt concentrations and driving salt consumption [5]. The obvious question is then: does ENaC play any role in sodium detection in humans? In this review, I will discuss current information supporting or arguing against the possible involvement of this ion channel in human salt taste. I will evaluate whether data from human studies fit the model for the detection of Na^+^ based on ENaC as sodium receptor localized at the apical membrane of taste cells; that is, where these cells contact the saliva in the so-called taste pore region of taste buds [6,7].

## 2. Psychophysics

In laboratory rodents, a pharmacological feature of taste ENaC is its sensitivity to amiloride, a diuretic drug, which selectively blocks the channel at submicromolar concentrations [8]. Since ENaC is inhibited by amiloride, application of this drug during stimulation with NaCl blunts the taste response and the attractiveness of low sodium concentrations [9,10]. Following the same methodological approach, the involvement of the ENaC pathway in human taste reception has been investigated over 15 years by sensory evaluation of the amiloride effect on salt taste. Unexpectedly, findings have been very controversial. Some authors found that indeed the presence of amiloride reduced the perceived saltiness of the NaCl solutions, although to varying degrees [11,12,13,14,15]. On the contrary, other investigators found that amiloride had negligible or no effect on salt taste [16,17,18,19,20,21]. The discrepancy among studies might be due to differences in the experimental design. For example, perception arising from stimulation of the anterior tongue seems to be affected by amiloride [11,12,13], whereas perception from whole-mouth stimulation is not [16]. This raises the possibility that in the whole-mouth protocol, additional sensory inputs from the oral cavity may “obscure” the information conveyed by amiloride-sensitive pathway at the level of central processing [22]. However, other studies in which stimuli were delivered only to the anterior dorsal surface of the tongue failed to find a significant effect of amiloride on saltiness perception [17,20,21]. Of note, amiloride strongly reduces the sour side taste of salt solutions when subjects can use not only one response category (saltiness), but all taste qualities (saltiness, sweetness, sourness, bitterness) to describe their perception [17,19].

Further factors affecting the outcome of the sensory assays might include the impact of amiloride bitterness in establishing the taste quality of salt solutions as well as the amiloride concentration tested. It is worth noting that some studies showing the amiloride effect used high drug concentrations (500 µM and 1 mM) [12,13,14], whereas studies claiming a negligible effect of amiloride on salt perception adopted smaller concentrations of the drug (10–100 µM) [17,18,20,21]. The specificity of amiloride for ENaC is true only for low drug concentration (half-maximal inhibition in submicromolar range; [8]). At higher concentrations, amiloride also affects other cellular proteins, including ion channels, transporters, and receptors [3,23]. A few examples of the molecular targets other than ENaC are shown in Table 1. It is worth noting the same proteins are also found in the taste cells of laboratory rodents (Table 1, rightmost column). As nicely pointed out by Lindemann [3] “if concentrations above 10 µM are needed for half-maximal inhibition, effects of amiloride other than blockage of Na^+^ channels need to be considered”. Thus, it is possible that human testing may have yielded contrasting results due to nonspecific effects of amiloride. However, it is also possible that ENaC in humans displays a lower sensitivity to amiloride than typical ENaC, as indicated by molecular biology studies (see below).

## 3. Electrophysiology

In laboratory animals, application of NaCl solution to the tongue mucosa produces a transepithelial current due to the movement of ions across the epithelium [9,34,35]. This current is believed to be sustained mainly by Na^+^ entering taste cells via the apical ENaC since amiloride strongly reduces it. Obviously, during NaCl stimulation, a voltage drop between mucosal and serosal side of the tongue develops, and this voltage drop can be measured with adequate instrumentation [9,34,35]. This approach has been applied to human volunteers to evaluate the contribution of the amiloride-sensitive pathway to the perceived saltiness. Electrophysiological recordings of lingual surface potential (LSP) in response to focal NaCl stimulation have provided evidence that, in some individuals, amiloride (100 μM) was able to reduce the voltage drop caused by NaCl application [36]. However, the effect was highly variable among individuals, ranging from 0% to 42% inhibition. Further studies demonstrated a positive correlation between LSP and the perceived intensity of saltiness [37]. It was also found that in expert salt tasters, amiloride (10 µM) impaired the ability to distinguish between two different salt concentrations (100 and 300 mM NaCl) [37]. Again, not all subjects exhibiting a LSP during NaCl stimulation were sensitive to amiloride, underscoring the high variability of the amiloride effect across individuals observed previously [36]. Nonetheless, these electrophysiological studies seem to confirm the psychophysical findings suggesting suppression of perceived saltiness by amiloride in some individuals when a small area of the tongue surface is stimulated [11,12,13].

## 4. Molecular Biology and Immunohistochemistry

In laboratory rodents, ENaC is an oligomeric protein made of three nonidentical subunits, named α-, β-, and γ-ENaC [38,39]. Expression cloning studies with *Xenopus* oocytes have clearly indicated that the α-subunit is required to induce channel activity, whereas the presence of the β- and γ-subunit allows maximal expression of sodium current [40]. Although it is still unknown how these subunits assemble to form ENaC in taste cells [41], all of them have been detected in taste tissues from laboratory animals by using molecular and immunohistochemical techniques [42,43,44,45,46]. It is important to underscore that there is also direct evidence that amiloride-sensitive taste cells do have ENaC subunits, whereas amiloride-insensitive cells do not [46]. By applying the same techniques on tissue samples from human subjects, it has been possible to establish that α-, β-, and γ-ENaCs occur in human taste papillae [47,48]. In man, however, taste tissues also express an additional δ-subunit, which is missing in rodents [48,49]. The δ-subunit is analog to the α-subunit in that its presence is necessary to form a Na^+^-permeable channel [50]. It is then possible that in human taste tissues, ENaC may include either an α- or δ-subunit. This subunit change may have an impact on the amiloride sensitivity, since replacement of α-subunit by a δ-subunit makes the channel 50-fold less sensitive to amiloride [50,51,52]. If this is the case, then the negligible effect of amiloride observed in some psychophysical studies (e.g., [17,18]) could be attributed to variations in the molecular composition of the ENaC protein [53]. It is worth noting that the expression level of ENaC subunits may vary significantly among subjects and that ENaC mRNAs are also detected in nontaste epithelium [48].

The detection of ENaC subunits in human taste tissues does not necessarily imply that this channel is involved in the initial events of sodium detection. According to the model of sodium taste detection proposed for rodents, ENaC should be found at the apical membrane of taste cells to work as a sodium receptor [3,8]. Immunohistochemical localization of ENaC subunits in human lingual epithelium has revealed that δ-ENaC is exclusively restricted to the taste pore region in both fungiform and circumvallate taste buds [48]. However, it has not been possible to establish whether this subunit localized to the apical membrane of taste cells or to tight junctions surrounding the apical ends of these cells. Unexpectedly, the other ENaC-subunits were found in the basolateral compartment of taste cells, which is involved in later stages of the sensory transduction and in intercellular communications with nerve endings and adjacent cells [5]. This raises the possibility that ENaC may serve other functions in taste buds. Recent findings indicate that ENaC plays a key role in the regulation of adult neurogenesis [54]. It is then tempting to speculate that ENaC might be involved in taste cell development since these cells continuously turnover [55,56]. Of note, another study found that δ-ENaC immunoreactivity was distributed over both the apical and basolateral ends of fungiform taste cells [49]. Although it is not clear how to reconcile these conflicting results on labeling pattern, both findings support the notion that δ-ENaC is expressed in human taste buds.

Although the model of sodium detection involving apically located ENaC explains several experimental observations, ENaC-subunits localized to the basolateral membrane could also mediate sodium reception by sensing Na^+^ leaked through tight junctions around taste cells [5]. Thus, this paracellular pathway could be responsible for activation of taste cells even in the absence of apical ENaC. Since the basolateral compartment of taste cells is exposed to an extracellular solution containing about 150 mM Na^+^, a significant diffusion of this cation through tight junctions could occur only if Na^+^ concentration in the mucosal surface is much higher than 150 mM. It is then possible that basolateral ENaCs may be relevant for sodium detection when salt concentration in the stimulating solution exceeds plasma tonicity. The basolateral localization of ENaC subunits may be a further factor in determining the variable amiloride sensitivity observed in human studies (see above).

## 5. Genetics

Single nucleotide polymorphisms (SNPs) in the gene coding for the ENaC β-subunit (*SCNN1B*) are somehow associated with changes in suprathreshold taste sensitivity for NaCl solutions, but not with salt taste threshold [57]. The β-subunit does not play a role in pore formation of the channel protein, but it is assumed to modulate channel activity and to be important for channel trafficking to the cell membrane [40,58,59]. Thus, these findings indicate that variations in the β-ENaC genes may contribute to differences in salt taste perception among individuals through a possible effect on the expression of ENaC in the taste cell membrane. Interestingly, these data imply that ENaC may be involved in the recognition of NaCl at concentrations that would have an impact on the actual consumption of dietary salt, that is, at suprathreshold concentrations. As pointed out by Contreras [60], in general, people do not add salt to food in order to be able to just detect it, but do so to a preferred suprathreshold level. It is noteworthy that amiloride tends to reduce suprathreshold intensities of perceived NaCl in adult volunteers [15]. However, a significant difference in taste intensity ratings between individuals with SNPs was found only for large concentrations of NaCl, such as 1 M [57]. Aqueous solutions of salt above ~150 mM are not preferred by humans [61]. In animals, salt levels exceeding tonicity of blood plasma are normally not accepted [62]. High salt concentrations activate other sensory pathways in addition to the ENaC-mediated one, including an amiloride-insensitive taste pathway and trigeminal nerve endings [5,22]. These components of salt reception mediate aversion responses and work as warning mechanisms to avoid the ingestion of hyperosmotic salt solutions [5,10,63]. Thus, the findings on SNPs of the ENaC β-subunit seemingly do not fit the model involving ENaC as low-salt receptor.

## 6. Salt Taste Enhancers

Chemicals able to increase the sensation evoked by NaCl without being salty themselves, the so-called “salt taste enhancers”, have attracted the attention of researchers for many years [64]. The reason is that these substances may be used to reduce salt content in processed foods to prevent excessive sodium intake, which is linked to the development of hypertension and subsequent pathologies [65,66]. Research on the mechanisms underlying the action of salt taste enhancers has provided some clues on the peripheral events leading to salt taste perception in humans.

Studies on human αβγ- or δβγ-ENaC functionally expressed in *Xenopus* oocytes have shown that sodium current through ENaCs is activated by salt-taste-modulating substances, such as L-arginine (Arg) [48]. By monitoring the changes of intracellular calcium levels in cultured human fungiform taste (HBO) cells, Xu et al. [67] found recently that some arginyl dipeptides, which proved to work as potent salty taste enhancers (up to 20% increase in perceived saltiness [68]), induced a significant increase in the number of cultured cells responding to NaCl. They also found that the effect required the presence of either α-ENaC or δ-ENaC. These results clearly indicate that salt taste enhancers target the human sodium receptor ENaC in both the αβγ or δβγ form. Unexpectedly, Arg was unable to stimulate cultured human taste cells [67], although it enhances the perceived saltiness in human sensory evaluations [48,68].

**Figure 1:**
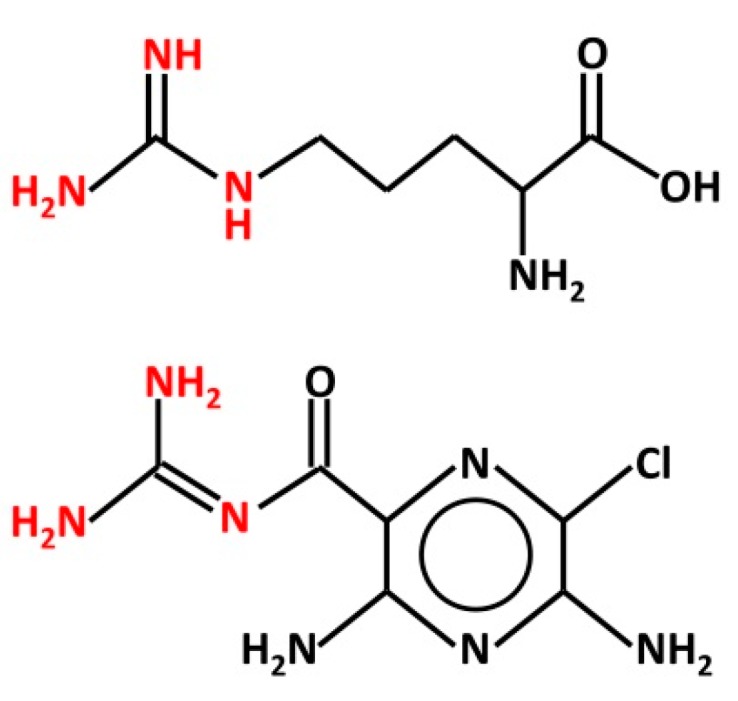
Structure of L-arginine (top) and amiloride (bottom). Both chemicals bear a guanidinium group (red), which is protonated in physiological conditions. This group is believed to interact with the ENaC channel pore from the extracellular space.

There are some aspects of the above studies that require keen consideration. In particular, it is remarkable how the effect of Arg may be affected by the cell system used to express human αβγ- or δβγ-ENaC. In *Xenopus* oocytes, Arg increases the sodium current through ENaCs [48], whereas in HBO cells, it is ineffective in changing intracellular calcium levels [67]. It is possible that this discrepancy may derive from the different experimental and methodological approach adopted. However, the finding that Arg potentiates ion currents through ENaC is, by itself, quite surprising. Both Arg and amiloride bear a guanidinium group (Figure 1), which occurs as a cation in physiological conditions (pH 7.4). It is believed that the positive charge-bearing guanidinium group of amiloride penetrates part of the ENaC channel pore, causing channel blockage, whereas the pyrazine group interacts with the outer mouth of the channel [69]. The chemical similarity may suggest that Arg, like amiloride, could affect ENaC directly from the extracellular space. Indeed, Ogawa et al. [70] suggested that “the guanidinium group of Arg may interact with sodium channels in taste bud membranes”. Yet, Arg enhances the current through ENaC, whereas amiloride reduces it.

## 7. Salivary Proteins

Proteins represent an important component of the saliva [71], the medium carrying Na^+^ to the apical, chemosensitive tips of taste cells. Recent studies on human subjects have found a correlation between salivary serine-type endoprotease activity and sensitivity to NaCl [72]. Since serine proteases increase the activity of ENaC through proteolytic cleavage [73,74], the authors have proposed that endoproteases of the saliva might affect salt taste sensitivity by modifying ENaC functioning. Stolle et al. [75] have identified a tetrapeptide that is likely released from salivary proteins by serine-type endoprotease activity and that is able to enhance salt taste perception. This means that in the saliva of salt sensitive subjects, an endogenous salt enhancer might be produced by enzymatic cleavage. It was also found that the abundance of two salivary proteins, lipocalin-1 and lysozyme C, could be related to individuals’ low sensitivity to NaCl. The authors have put forward the hypothesis that electrostatic interaction of these proteins with ENaC in taste cells may reduce the accessibility of sodium ions to ENaC [75]. These findings are clearly fascinating, but do not prove that ENaC actually functions as a sodium receptor in humans.

## 8. Discussion

The possible involvement of the ion channel, ENaC, in human taste reception has been investigated with different approaches. To date, however, it is not possible to provide a definitive answer as to the role of the ENaC pathway in producing salty sensations due to inconsistent findings. Perhaps the more conflicting results are from psychophysical studies involving the use of amiloride, a pharmacological probe for ENaC, to challenge saltiness perception. There are several issues regarding the adopted methodology that might be responsible for the observed discrepancies. For instance, the amiloride concentration used in sensory tests is not always adequate to avoid side effects on other ion channels and transporters. Thus, the apparent effect of amiloride in some studies (e.g., [12,13,14]) might be somehow misleading. Nonetheless, electrophysiological studies suggest that the application of salt solution on the human tongue induces a voltage drop across the mucosa that is similar to the one observed in laboratory animals. However, ENaC mRNAs are found also in nontaste epithelium [44,48,76], raising the possibility that the ion current crossing the mucosa could also be due to Na^+^ diffusion through epithelial cells.

According to the model proposed for rodents, ENaC should be found at the apical membrane of taste cells, which stick out into the taste pore bathed by saliva containing taste stimuli [3,8]. Available data suggest that only the δ-subunit localizes to the taste pore region in human taste buds, whereas other ENaC subunits seem to be segregated in the basolateral compartment, beneath the apical tips of taste cells. It is not yet known whether all the subunits are required to form a functional sodium receptor [41]. Differential expression of ENaC-subunits has been described in transporting epithelia [77], suggesting that endogenous channel in vivo may require only one or two subunits to work properly. δ-ENaC expressed alone in *Xenopus* oocytes is able to mediate a membrane current [50]. Thus, the occurrence of the δ-subunit in the taste pore region of human taste buds seems to suggest that it may function as a sodium receptor. Unfortunately, the microscopic analysis has not allowed establishing with confidence whether this subunit lies in the apical end of taste cells or in the tight junctions connecting adjacent taste cells just below the taste pore [48]. Indirect evidence supporting a role for ENaC in human salt taste has been provided by genetic studies [57] and by in vitro assays on cells expressing the human αβγ- or δβγ-ENaC [48,67], although there is some inconsistency among these data. Recent analysis of the correlation between the salivary proteome and the salt sensitivity in human volunteers are seemingly consistent with a role of ENaC [72,75].

In conclusion, the available data are suggestive of possible involvement of ENaC in human sodium detection, although it is not clear whether this occurs at the beginning of the reception process (interaction between sodium receptor and Na^+^ at the apical membrane of taste cells) or later on, after Na^+^ has been detected. The lack of the amiloride effect in some psychophysical studies [16,17,18,20,21] and the presence of α-, β-, and γ-subunit only in the basolateral portion of taste buds [48] seem to favor a role for ENaC downstream of the initial receptive events. Consistent with this hypothesis is the finding that SNPs in the gene coding for the ENaC β-subunit affect suprathreshold sensitivity to salt solutions, that is, at concentration levels above the detection/recognition threshold [57]. Clearly, further research is required to obtain a coherent and thorough comprehension of the early events of sodium detection in human taste cells. This information represents the premise for understanding interindividual variability in the function of sodium taste receptors and its potential implications for eating behavior.

## Figures and Tables

**Table 1:** Molecular targets, other than ENaC, for the inhibitory effect of amiloride expressed by the inhibition constant (*K_i_* = amiloride concentration producing 50% inhibition). Data refer to nontaste tissues. The occurrence of these molecular targets in rodents taste cells is also referenced.

Molecular Target	*K_i_* (µM)	Cell/Tissue	Occurrence in Rodent Taste Cells
T-type calcium channel	30	Mouse neuroblastoma and chick DRG ^1^ neurons [24]	[25,26]
Na^+^/H^+^ exchanger	30	Rabbit renal microvillous membrane [27]	[28,29,30]
Muscarinic receptors	40–80	Rabbit pancreatic acini [31]	[32,33]

^1^ Dorsal Root Ganglion.
